# Supplementary material for: Characterization of Sugar Reduction in Model Confectionary Gels Using Descriptive Analysis
Source: Gels. 2022 Oct 11;8(10):644. doi: 10.3390/gels8100644 (PMC9601666; doi:10.3390/gels8100644)
Supplement: Supplementary file 1 [file gels-08-00644-s001.zip › gels-1946035-supplementary.pdf]

Table S1. Instrumental and Sensorial Definitions of Texture Profile Analysis (TPA) parameters [12,31,46]

Parameter	Instrumental Definition	Sensory Definition
Hardness	The peak force during the first compression cycle	Force required to compress food between the molars
Springiness	The height that the food recovers during the time that elapses between the end of the first bite and the start of the second bite	The extent to which a compressed food returns to its original shape
Adhesiveness	the negative force area for the first bite representing the work necessary to pull the plunger away from the food sample	The work required to pull the food away from the mouth during the eating process
Cohesiveness	the ratio of positive force area during the second compression portion to that during the first compression (Area 2/Area1)	The strength of the internal bonds making up the food; the degree a substance is compressed before it breaks
Fracturability	The force at the first significant break in the curve	The force at which the material fractures, crumbles, cracks or shatters
Chewiness	The product of gumminess and springiness	The energy required to chew a solid food until it is ready for swallowing
Gumminess	The product of hardness and cohesiveness	The energy required to disintegrate a semisolid food so that it is ready for swallowing
